# Repeatability of [^18^F]FDG PET/CT total metabolic active tumour volume and total tumour burden in NSCLC patients

**DOI:** 10.1186/s13550-019-0481-1

**Published:** 2019-02-07

**Authors:** Guilherme D. Kolinger, David Vállez García, Gerbrand M. Kramer, Virginie Frings, Egbert F. Smit, Adrianus J. de Langen, Rudi A. J. O. Dierckx, Otto S. Hoekstra, Ronald Boellaard

**Affiliations:** 1 University of Groningen, University Medical Center Groningen, Department of Nuclear Medicine and Molecular Imaging, Hanzeplein 1, 9713 GZ Groningen, The Netherlands; 2 Amsterdam University Medical Centers, location VU Medical Center, Department of Radiology and Nuclear Medicine, De Boelelaan 1117, 1081 HV Amsterdam, The Netherlands; 3 Amsterdam University Medical Centers, location VU Medical Center, Department of Pulmonary Disease, De Boelelaan 1117, 1081 HV Amsterdam, The Netherlands; 4 Netherlands Cancer Institute, Department of Thoracic Oncology, Plesmanlaan 121, 1066 CX Amsterdam, The Netherlands

**Keywords:** Repeatability, Total tumour burden, Metabolic active tumour volume, FDG PET/CT, NSCLC, Tumour delineation, Tracer uptake interval, Majority vote

## Abstract

**Background:**

Total metabolic active tumour volume (TMATV) and total tumour burden (TTB) are increasingly studied as prognostic and predictive factors in non-small cell lung cancer (NSCLC) patients. In this study, we investigated the repeatability of TMATV and TTB as function of uptake interval, positron emission tomography/computed tomography (PET/CT) image reconstruction settings, and lesion delineation method. We used six lesion delineation methods, four direct PET image-derived delineations and two based on a majority vote approach, i.e. intersection between two or more delineations (MV2) and between three or more delineations (MV3). To evaluate the accuracy of those methods, they were compared with a reference delineation obtained from the consensus of the segmentations performed by three experienced observers. Ten NSCLC patients underwent two baseline whole-body [^18^F]2-Fluoro-2-deoxy-2-D-glucose ([^18^F]FDG) PET/CT studies on separate days, within 3 days. Two scans were obtained on each day at 60 and 90 min post-injection to assess the influence of tracer uptake interval. PET/CT images were reconstructed following the European Association of Nuclear Medicine Research Ltd. (EARL) compliant settings and with point-spread-function (PSF) modelling. Repeatability between the measurements of each day was determined and the influence of uptake interval, reconstruction settings, and lesion delineation method was assessed using the generalized estimating equations model.

**Results:**

Based on the Jaccard index with the reference delineation, the MV2 lesion delineation method was the most successful method for automated lesion segmentation. The best overall repeatability (lowest repeatability coefficient, RC) was found for TTB from 90 min of tracer uptake scans reconstructed with EARL compliant settings and delineated with 41% of lesion’s maximum SUV method (RC = 11%). In most cases, TMATV and TTB repeatability were not significantly affected by changes in tracer uptake time or reconstruction settings. However, some lesion delineation methods had significantly different repeatability when applied to the same images.

**Conclusions:**

This study suggests that under some circumstances TMATV and TTB repeatability are significantly affected by the lesion delineation method used. Performing the delineation with a majority vote approach improves reliability and does not hamper repeatability, regardless of acquisition and reconstruction settings. It is therefore concluded that by using a majority vote based tumour segmentation approach, TMATV and TTB in NSCLC patients can be measured with high reliability and precision.

**Electronic supplementary material:**

The online version of this article (10.1186/s13550-019-0481-1) contains supplementary material, which is available to authorized users.

## Background

Quantitative evaluation of cancer therapy response is an essential step towards effective and personalised patient treatment. Positron emission tomography (PET) combined with computed tomography (CT) using [^18^F]2-Fluoro-2-deoxy-2-D-glucose ([^18^F]FDG) is a powerful tool to provide predictive information on treatment response in non-small cell lung cancer (NSCLC) patients [1–4]. Despite the availability of a diversity of metrics that can be derived from [^18^F]FDG PET/CT images, treatment response is usually measured using the change in standardised uptake values (SUV) [5–8], even though SUV is sensitive to a series of patient and scan protocol factors and is only accurate when there is homogeneous uptake in the tumour [9–12]. As a result, interest in different quantitative features has been growing and, rather than evaluating individual lesions, there is a shift towards metrics that better represent the patient’s total tumour load, such as the total metabolic active tumour volume (TMATV) and total tumour burden (TTB), also referred to as whole-body total lesion glycolysis (TLG) [13–17]. TMATV, for example, has been found to be a significant prognostic factor for disease progression, recurrence, and death [16, 18]. TTB combines volumetric and metabolic information to represent whole-body disease burden and is regarded as a strong prognostic indicator for NSCLC, which can be important when defining treatment guidelines [13]. Despite this increase in interest on whole-body metrics, the majority of tumour test-retest studies only evaluated the repeatability of SUV and primarily on lesion basis, which were summarised by Lodge [19].

All quantitative measurements from [^18^F]FDG PET/CT scans are affected by tracer uptake time and image reconstruction settings [15, 20, 21]. To this end, the European Association for Nuclear Medicine Research Ltd. (EARL) has developed procedure guidelines for [^18^F]FDG PET/CT tumour imaging to improve standardisation of uptake values in multicentre settings [22]. On the other hand, modern reconstructions include resolution modelling based on the PET/CT system point-spread-function (PSF) [23, 24] and are considered state-of-the-art in clinical practice due to its higher resolution and improved visual lesion detection. However, use of PSF affects the metrics derived from PET images [21, 25] and it is, at present, not compliant with the current standardisation proposed by European Association for Nuclear Medicine (EANM) guidelines. Consequently, there is a high interest in exploring the quantitative features extracted from PSF-reconstructed PET images and to compare them with EARL compliant metrics [25]. Of note, recently the feasibility of performance harmonisation using state-of-the-art PET/CT systems was shown, enabling the use of PSF reconstruction in multicentre studies [26].

Moreover, there are many lesion delineation methods, all of which are influenced by scan and reconstruction parameters; hence, metrics that depend on the estimated lesion volume such as TMATV and TTB are also affected [14, 25, 27]. To address this performance variability, it can be expected that a tumour delineation based on the agreement of several delineation methods will improve the reliability of the lesion segmentation against image quality variations [28].

Therefore, the aim of this study is to assess the repeatability of TMATV and TTB from whole-body [^18^F]FDG PET/CT scans of NSCLC patients and to investigate its sensitivity to image acquisition, reconstruction settings, and lesion delineation method, including methods based on the majority vote approach.

## Methods

### Patients

Ten NSCLC patients underwent a total of four baseline whole-body [^18^F]FDG PET/CT scans on two different days, within 3 days. At each day, scans were obtained at both 60 and 90 min post-injection. The scan at 90 min post-injection of one patient on the second day was excluded due to excess movement. Another patient could not undergo the scan at 90 min on the second day. Further patient information and inclusion criteria can be found in more detail in previous publications [15, 20]. A subset from that data was used on the present study since one patient from that dataset did not perform any scan on the second day and was excluded. Demographics of the patients are described in Table 1. All patients gave written informed consent before enrolment, and the study was approved by the Medical Ethics Review Committee of the VU University Medical Center (Dutch trial register [trialregister.nl] NTR3508).

**Table 1:** Patient demographics and scan characteristics

Characteristic	Overall	Scans at day 1	Scans at day 2
Patients	10		
Gender ratio (M/F)	1.5		
Age (years)	61 [45–66]		
Stage			
IIIB	3		
IV	7		
Histology			
Adenocarcinoma	7		
Squamous cell carcinoma	3		
Weight (kg)		76 [57–110]	75 [57–113]
Injected activity (MBq)		248 [194–377]	238 [192–392]
Scan start time (min)			
Uptake time goal of 60 min		61 [59–67]	60 [60–63]
Uptake time goal of 90 min		92 [90–97]	90 [90–95]

### [^18^F]FDG PET/CT acquisition and imaging processing

All PET/CT scans were obtained with a Gemini TF PET/CT scanner (Philips Healthcare, Cleveland, OH, USA). Patients fasted for 6 h or more. A low-dose CT during normal breathing for attenuation correction was performed, followed by a whole-body [^18^F]FDG PET scan 60 min after tracer injection. Thirty minutes later, a second whole-body PET acquisition was performed. After the second PET scan, a second low-dose CT was done for attenuation correction. This procedure was repeated within 3 days of the first study. All PET data were normalised and corrected for scatter and random events, dead time, attenuation, and decay. Two reconstruction protocols were applied to the PET images. The first reconstruction followed EARL compliant guidelines for tumour imaging [22], while the second included resolution modelling with PSF [23, 24] as implemented by the scanner vendor.

### Standard PET-based delineation methods

All images were segmented with four commonly used and readily available (including in clinical software tools) semi-automatic delineation methods [20, 22, 27, 29] with an in-house developed software. The tumour’s contours were defined by:

1. Fixed SUV threshold of 2.5 g/mL (SUV25)
2. Fixed SUV threshold of 4.0 g/mL (SUV40)
3. Adaptive at 41% of each lesion’s maximum SUV (41MAX)
4. Contrast corrected for local tumour to background activity at 50% of the peak SUV (A50P)

Note that SUV25 and SUV40 are simple methods based on fixed SUV threshold, 41MAX is adaptive to each lesion’s condition, drawing a mask at 41% of its SUV_max_ without regard to background activity, and A50P adaptively corrects for source to local background activity ratio and the method is able to segment lesions also in case tumour uptake would be lower than twice the local background. Local background activity was defined as a single-voxel 3D shell around each masked region, 2.5 cm away from the edges of an isocontour defined at 70% of the SUV_max_ value, excluding voxels with a value higher than 2.5. The mean uptake of this shell was considered the reference value for the local background activity [27]. The peak SUV was defined as a 1 mL sphere volume of interest with the highest SUV average across all positions within a lesion [29].

### Consensus contours

In addition to these four PET image-based delineation methods, two consensus contours were drawn using a majority vote (MV) approach. These consensus methods were based on the intersection of the four above-mentioned PET-based delineations, i.e. if a number of methods agree that a voxel is part of the lesion, then it will also be included in the consensus delineation:

1. MV2: Agreement between 2, 3, or 4 of the standard PET-based methods
2. MV3: Agreement between 3 or 4 of the standard PET-based methods

### Expert observer delineations

Images from the first day, acquired 60 min post-injection and reconstructed following EARL settings were assessed by three experienced observers (AB, RB, WN). The observers were blind to these conditions and did not know what images were being assessed. These images were chosen for their compliance with EANM Guidelines for NSCLC studies [22]. The observers performed segmentations assisted by the same in-house developed software used for the semi-automatic delineations. A whole-body automatic delineation of all [^18^F]FDG avid regions of the PET images was drawn using the SUV40 method, then the observers had to remove any region they considered to be physiological uptake and not a lesion. Next, the observers could add any region perceived as lesion that was missed by the automatic method. The SUV threshold for the delineations was adjusted with a slider, fine-tuning the segmentation of all regions at once. This procedure was repeated after 12 (AB), 7 (RB), and 13 (WN) days with images from the second day of scans (again 60 min post-injection scan; EARL compliant reconstruction). It was then possible to address the repeatability of the observers. Most importantly, the intersection of these delineations was evaluated at each day and, with a consensus approach, a reference delineation (RD) was created for each day: RD1 and RD2, respectively.

### Metrics

PET images were analysed with the six semi-automatic delineation methods. Therefore, each patient had 4 scans × 2 reconstructions × 6 semi-automatic segmentations = 48 possibilities studied. Additionally, the experienced observers and reference delineations were studied. For each possibility, the total segmented volume, summed over all lesions, was measured as TMATV. Furthermore, TLG was calculated per lesion as the MATV multiplied by its average SUV (SUV_mean_). The TTB of a patient is thus defined as the whole-body TLG, i.e. the sum of TLG over all lesions.

### Delineation success of semi-automatic methods

The six semi-automatic delineation methods were compared against the reference delineation obtained from the expert observers. The Jaccard index (JI) between the TMATV from the RD and each semi-automatic method was calculated for each scan day, only for images acquired 60 min post-injection and reconstructed with EARL compliant settings. A JI of 1.0 represents a perfect coincidence of volumes, while an index of 0.0 means there is no intersection between the two volumes. The JI between volumes A and B is defined as follows:

$$ \mathrm{JI}\left(A,B\right)=\frac{A\cap B}{A\cup B} $$

### Repeatability analysis

The repeatability (or test-retest) of TMATV and TTB were determined by the difference and relative difference between the values measured at each day. This was done for each combination of tracer uptake interval, reconstruction settings, and delineation method. The test-retest (TRT) was calculated as follows:

$$ \mathrm{TRT}=\mathrm{day}2-\mathrm{day}1 $$

$$ \mathrm{TRT}\%=100\times \frac{\mathrm{day}2-\mathrm{day}1}{\left(\mathrm{day}1+\mathrm{day}2\right)/2} $$

where day1 and day2 are the metrics (TMATV or TTB) determined at the same time point on both days. Following, the absolute of TRT and TRT% were also computed and indicated as aTRT and aTRT%. Additionally, intraclass correlation coefficients (ICC) were calculated to assess the agreement between the measurements at each day (two-way mixed model; consistency type; single measures).

The repeatability coefficient (RC) was calculated as the standard deviation (SD) of the respective TRT and TRT% of each combination of uptake interval, reconstruction settings, and lesion delineation (10 patients per combination) multiplied by 1.96:

$$ \mathrm{RC}=1.96\times \mathrm{SD} $$

According to previous literature, the mean difference ± RC provides an interval within which 95% of the differences between measurements of two consecutive measurements are expected to lie [19, 30].

### Statistical analysis

In order to study the effects of reconstruction settings, tracer uptake time, and delineation method on the repeatability of TMATV and TTB, the present data was analysed using the generalized estimating equations (GEE) statistical model [31–33]. The GEE model is known to achieve higher statistical power with small sample sizes, repeated measurements, and with missing data than the repeated measures ANOVA [32], and its known to be less affected by violations on the distribution assumption, as it only requires the correct specification of marginal mean and variance as well as the link function [33]. The best working correlation matrix, based on the quasi-likelihood under the independence model information criterion values was the exchangeable matrix, and an identity link function was used. The Wald test was used to report the *p* values, and *p* < 0.05 was considered significant, without correction for multiple comparisons.

To assess the differences between the repeatability of the semi-automatic delineation methods and how they were affected by tracer uptake time and image reconstruction settings, their repeatability (as TRT%) were included in the GEE model as dependent variables, and the patient number, tracer uptake interval, reconstruction settings, and delineation method were included as predictors (i.e. independent variables) for the model, as well as their interactions (with the exception of the interactions with the patient number, as this variable was included in the model to account for the missing data).

The ICC and the GEE statistical analyses were carried out using the SPSS software package (version 23.0, IBM, Armonk, NY, USA). Results are presented as mean difference ± standard error, unless mentioned differently.

## Results

### TMATV and TTB values distribution

**Fig. 1:**
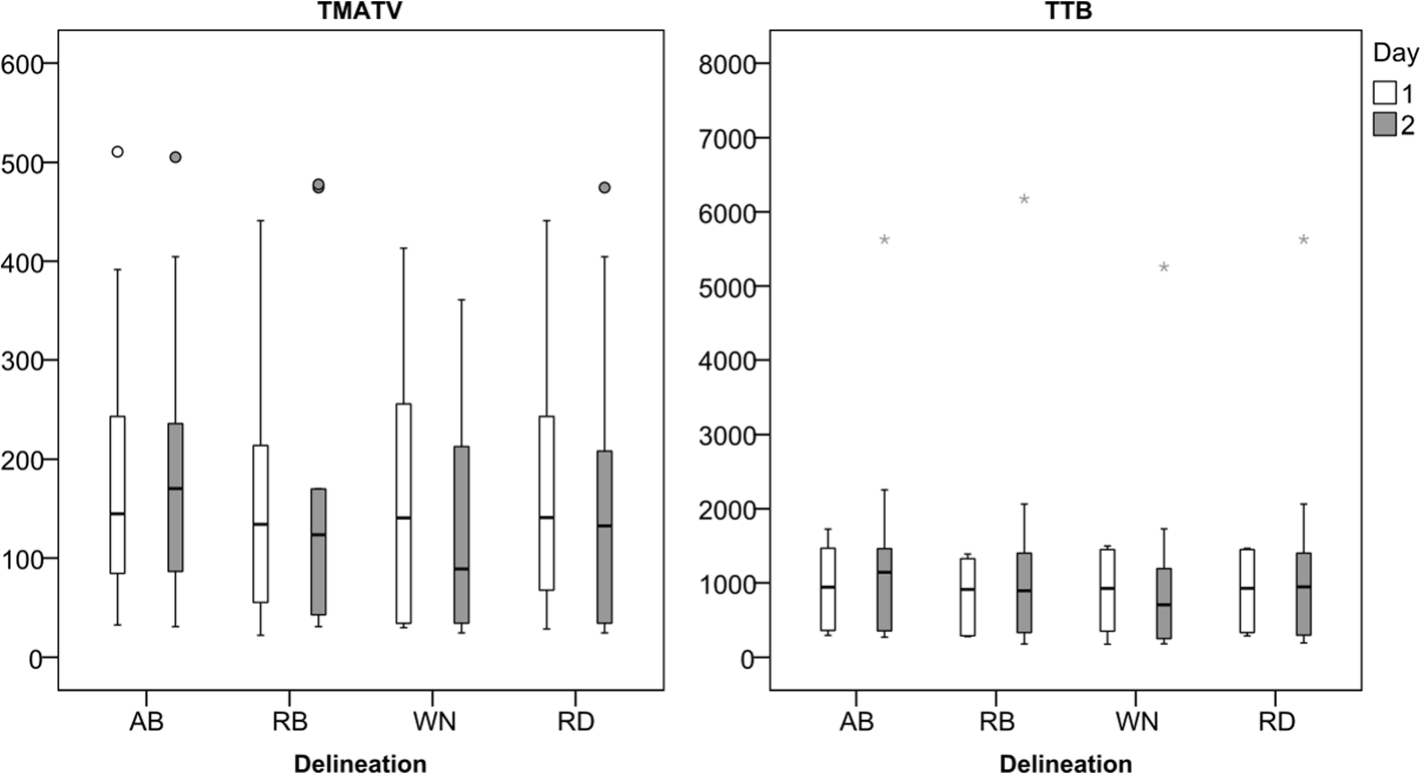
Box plots with median and range of TMATV (left panel) and TTB (right panel). Both panels show data from the delineations in images acquired at the first and second day of scans, indicated by the colours. Images were acquired 60 min post-injection and reconstructed following EARL compliant settings. TMATV in milliliter and TTB in grams

**Fig. 2:**
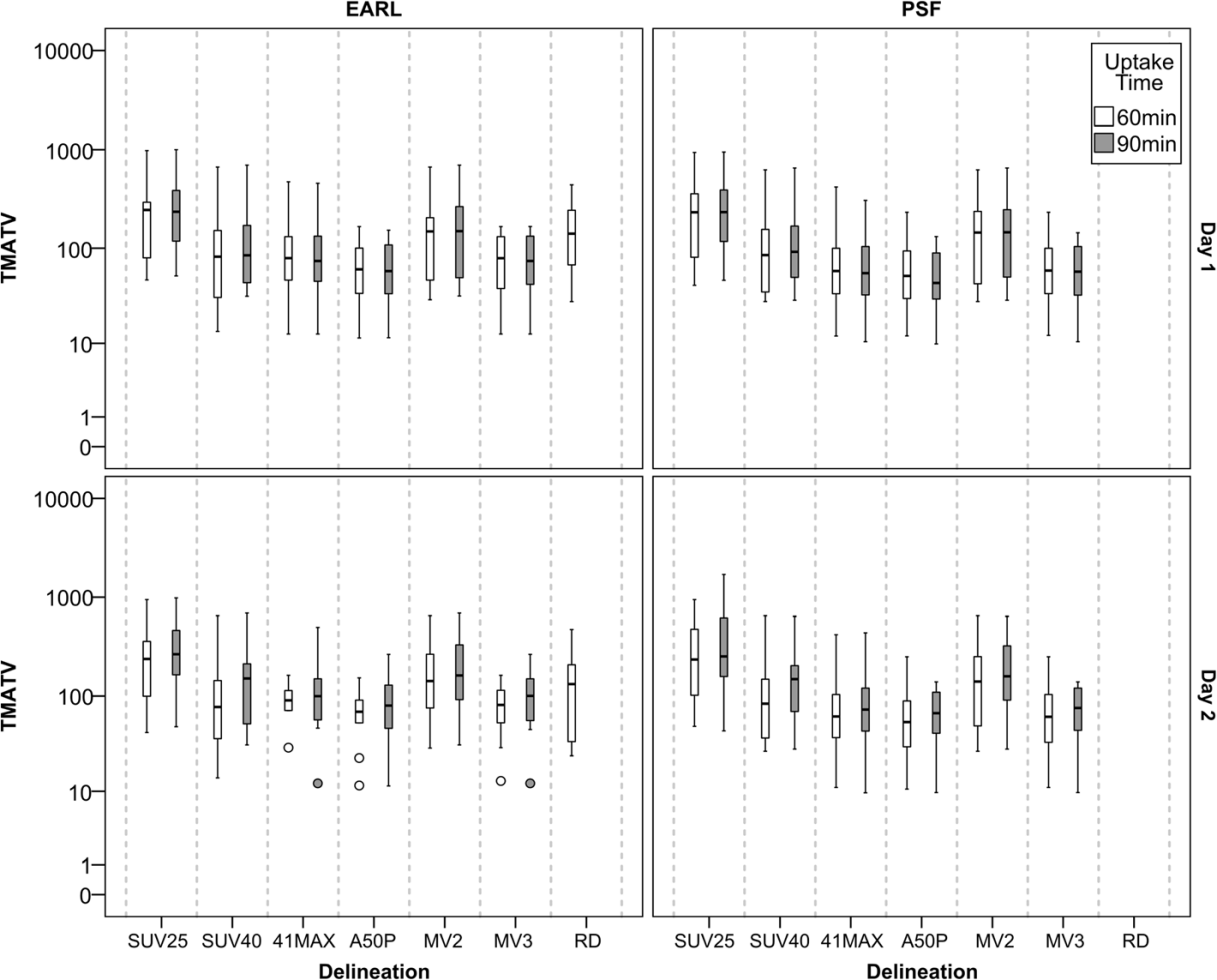
Box plots with median and range of TMATV (mL). *Y*-axis in log-10 scale for better visualisation; outliers included. Panels on the left show the values acquired from EARL compliant reconstruction by the six semi-automatic delineation methods as well as the reference delineation (horizontal axis). On the right panels, data from images with PSF reconstruction. On the top row, data from the test (day 1 of scans) is displayed, while on the bottom row from retest (day 2 of scans). Data from scans acquired 60 or 90 min post-injection are colour-coded

Scan protocol, reconstruction settings, and delineation method affected the metrics acquired from the [^18^F]FDG PET/CT scans. Therefore, the median and range of the data acquired for the patients will vary case by case. The data from the expert delineations and the reference can be seen in Fig. 1 for TMATV and TTB. Note the variability between observers, especially the median value (black horizontal line inside the box). Furthermore, RD compensates some of the inter-observer variability and its median values are between the values from individual observers. The TMATV acquired from the semi-automatic delineation methods can be seen in Fig. 2 (together with RD for comparison). The plot is displayed in a log-scale for better visualisation, since there is a large spread of the data (e.g. mainly small volumes in most of the tumours, with few cases with extremely large volumes). It was possible to observe that fixed SUV threshold methods (SUV25 and SUV40) segmented, in general, larger volumes than 41MAX and A50P. As a natural consequence, MV2 presents larger volumes than MV3. Additionally, Fig. 2 illustrates the effect of a longer tracer uptake interval on TMATV, resulting in larger volumes. This effect is more pronounced on the standard PET-based delineation methods than on consensus methods.

### Delineation accuracy

**Fig. 3:**
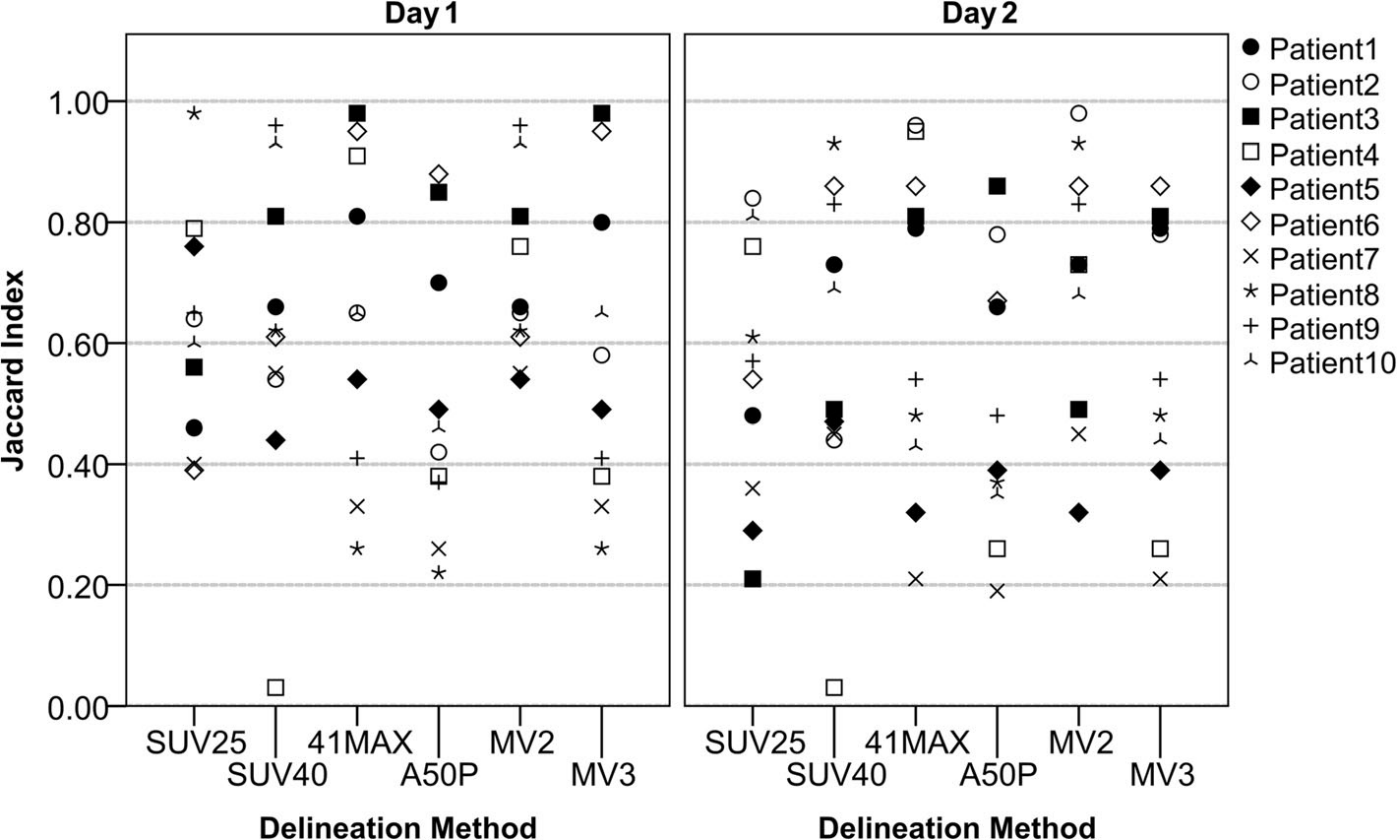
Jaccard index for all patients delineated with the semi-automatic delineation methods (when compared against RD) for day 1 (left panel) and day 2 (right panel). Each patient is displayed with a different symbol

The Jaccard index of each of the six semi-automatic delineation methods (when compared with RD1 and RD2) can be seen on Fig. 3 (images acquired at 60 min post-injection, and reconstructed with EARL compliant settings). Each patient is displayed with a different symbol, illustrating the varying JI scores of each delineation method and that no method was consistently the worst or the best for different patients. This is a consequence of the fact that a certain semi-automatic delineation method might be accurate for one patient while failing to delineate another patient, even under the same image settings. Figure 3 also demonstrates that MV2’s JI were, in general, higher than the scores from other methods. Table 2 shows the average JI and its interquartile range of the semi-automatic delineation methods (with RD1 and RD2).

**Table 2:** Jaccard index and interquartile range

Delineation method	Day 1		Day 2	
	Average JI	Interquartile range	Average JI	Interquartile range
SUV25	0.62	[0.48–0.73]	0.55	[0.39–0.72]
SUV40	0.62	[0.54–0.77]	0.59	[0.46–0.80]
41MAX	0.65	[0.45–0.88]	0.64	[0.45–0.85]
A50P	0.50	[0.37–0.65]	0.50	[0.35–0.67]
MV2	0.71	[0.61–0.80]	0.70	[0.54–0.86]
MV3	0.58	[0.39–0.77]	0.56	[0.40–0.79]

Average Jaccard index and the interquartile range of each semi-automatic delineation method. JI with the reference delineation of each day, RD1 and RD2, respectively. For scans acquired 60 min after injection and reconstructed following EARL compliant settings

The consensus contour MV2 has the highest average score for both days (0.71 and 0.70) and the smallest interquartile range (0.18 and 0.32). Following, the second highest average JI was obtained with 41MAX (0.64 and 0.65) and the method with the lowest average score was A50P (0.5). Some examples of the delineations on images acquired 60 min post-injection and reconstructed with EARL compliant settings can be seen in Additional file 1: Figure S1.

Additional file 1: Table S1 presents the average JI and its interquartile range of the semi-automatic delineation methods applied to both EARL and PSF reconstruction settings, as well as for images acquired 90 min post-injection.

### Repeatability: experienced observers

**Fig. 4:**
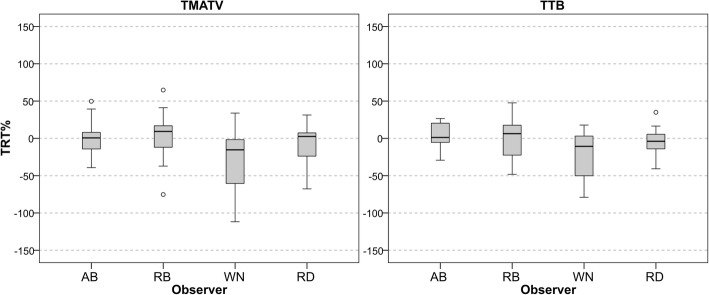
Box plots of TMATV (left panel) and TTB (right panel) repeatability from the three experienced observers and their consensus, RD

The repeatability of each experienced observer and of the RD can be seen in Table 3, where the average (from the 10 patients) TRT, TRT%, their respective RC, and ICC values are displayed for TMATV and TTB. Figure 4 shows the box plots for TRT% of each experienced observer and of RD. Observer’s repeatability was low, with up to 50% TMATV variation. Furthermore, this assessment was highly dependent on the observer and a consensus between the expert observers lowered TRT% variability (Table 3).

**Table 3:** Repeatability of experienced observers and reference delineation

Observer	TMATV			TTB		
	TRT (RC)	TRT% (RC%)	ICC	TRT (RC)	TRT% (RC%)	ICC
AB	4.2 (98)	1.6 (53)	0.95	83 (426)	3.2 (37)	0.99
RB	4.6 (73)	2.5 (76)	0.97	94 (631)	− 0.5 (54)	0.98
WN	− 43.7 (161)	− 28.6 (86)	0.79	− 121 (648)	− 19.1 (66)	0.98
RD	− 5.7 (82)	− 9.8 (61)	0.96	41 (505)	− 4.6 (43)	0.99

Average repeatability and repeatability coefficient and corresponding ICC of total metabolic active tumour volume (TMATV) and total tumour burden (TTB) for the three experienced observers and the reference delineation. TRT in mL and TRT% in percentage

### Repeatability: semi-automatic delineation methods

A summary of repeatability, RC, and ICC for all semi-automatic delineation methods applied to both uptake intervals and reconstructions can be found in Tables 4 and 5 for TMATV and TTB, respectively (RD is shown together for comparison). The best overall repeatability, as defined by the lowest RC%, was found for TTB derived from 90 min post-injection scans with EARL compliant reconstruction and 41MAX delineation method (RC = 11%). Furthermore, the same method under the same parameters had the best TMATV repeatability (RC = 14.3%). The summary for aTRT and aTRT% for both TMATV and TTB are shown in Additional file 1: Table S2 and Table S3, in which the lowest RC values are 8.9% for TMATV (60 min of uptake, PSF reconstruction, A50P delineation) and 5.5% for TTB (90 min of uptake, EARL compliant reconstruction, 41MAX delineation).

**Table 4:** Total metabolic active tumour volume repeatability for different tracer uptake intervals, reconstruction settings, and lesion delineation methods

Method	60 min of uptake			90 min of uptake		
	TRT (RC)	TRT% (RC%)	ICC	TRT (RC)	TRT% (RC%)	ICC
EARL						
SUV25	29.7 (211)	5.9 (51)	0.93	9.4 (35)	3.7 (15)	1.00
SUV40	2.5 (44)	1.0 (31)	0.99	3.5 (17)	7.9 (32)	1.00
41MAX	4.5 (35)	7.8 (45)	0.99	6.5 (30)	3.0 (14)	1.00
A50P	0.4 (38)	5.9 (49)	0.92	16.2 (77)	10.5 (37)	0.83
MV2	9.4 (48)	9.3 (44)	0.99	5.4 (24)	3.5 (15)	1.00
MV3	− 4.2 (36)	1.2 (39)	0.94	15.7 (78)	9.1 (38)	0.83
RD	− 5.7 (82)	− 9.8 (61)	0.96			
PSF						
SUV25	46.0 (171)	14.9 (44)	0.95	139.5 (738)	14.4 (63)	0.65
SUV40	3.4 (23)	1.8 (29)	1.00	3.8 (19)	5.6 (22)	1.00
41MAX	3.2 (17)	6.9 (35)	1.00	17.4 (92)	6.2 (30)	0.92
A50P	2.1 (15)	0.2 (19)	0.99	5.7 (19)	8.2 (21)	0.98
MV2	5.4 (25)	3.0 (35)	1.00	14.5 (75)	5.6 (22)	0.98
MV3	2.8 (14)	3.1 (18)	1.00	3.7 (20)	3.9 (21)	0.98

Average and repeatability coefficient of total metabolic active tumour volume (TMATV) repeatability for different tracer uptake intervals, reconstruction settings, and lesion delineation methods, including the corresponding ICC. TRT in mL and TRT% in percentage

**Table 5:** Total tumour burden repeatability for different tracer uptake intervals, reconstruction settings, and lesion delineation methods

Method	60 min of uptake			90 min of uptake		
	TRT (RC)	TRT% (RC%)	ICC	TRT (RC)	TRT% (RC%)	ICC
EARL						
SUV25	322 (1560)	5.8 (56)	0.95	59 (319)	4.0 (18)	1.00
SUV40	236 (1149)	3.7 (54)	0.97	38 (252)	8.4 (34)	1.00
41MAX	122 (611)	8.9 (47)	0.93	59 (169)	3.1 (11)	1.00
A50P	126 (667)	7.6 (56)	0.90	92 (287)	9.7 (36)	0.99
MV2	257 (1133)	9.4 (54)	0.97	44 (257)	4.1 (21)	1.00
MV3	96 (633)	3.7 (48)	0.93	91 (282)	8.4 (35)	0.99
RD	41 (505)	− 4.6 (43)	0.99			
PSF						
SUV25	309 (1069)	9.1 (45)	0.98	462 (2490)	10.0 (41)	0.93
SUV40	133 (780)	1.2 (39)	0.99	41 (260)	6.3 (26)	1.00
41MAX	65 (326)	3.1 (38)	0.98	83 (272)	5.9 (22)	0.99
A50P	60 (355)	− 1.2 (32)	0.97	64 (182)	8.6 (28)	1.00
MV2	138 (778)	1.4 (42)	0.99	70 (333)	5.7 (24)	1.00
MV3	62 (322)	0.8 (28)	0.98	44 (115)	4.5 (17)	1.00

Average and repeatability coefficient of total tumour burden (TTB) repeatability for different tracer uptake intervals, reconstruction settings, and lesion delineation methods including the corresponding ICC. TRT in mL and TRT% in percentage

All TMATV ICCs are higher than 0.90, except for A50P and MV3 applied to EARL compliant images at 90 min post-injection (both with ICC = 0.83), and SUV25 applied to PSF images at 90 min post-injection (ICC = 0.65). TTB had overall higher ICC than TMATV, with values equal to or higher than 0.90 for all delineation methods, regardless of uptake interval and image reconstruction settings.

### Repeatability: impact of tracer uptake interval

The overall effect of tracer uptake time on TMATV (− 1.34% ± 4.49%; *p* = 0.766) and TTB (2.14% ± 5.57%; *p* = 0.701) repeatability was not significant. TMATV repeatability of specific delineation methods and reconstruction settings was not affected by changes in tracer uptake interval. Similarly, TTB repeatability was not affected by using a specific reconstruction and delineation method.

### Repeatability: impact of reconstruction settings

Changes in reconstruction settings were not a significant factor impacting TMATV (− 0.43% ± 1.96%; *p* = 0.827) and TTB (1.78% ± 2.22%; *p* = 0.422) repeatability. Repeatability of metrics from scans at different uptake intervals was not significantly affected by different reconstructions. However, the SUV25 delineation method had significantly different TMATV repeatability with different reconstructions (− 9.87% ± 4.19%; *p* = 0.018), while other methods did not (*p* ≥ 0.300). Nevertheless, at specific uptake interval using a certain delineation method, changes in reconstruction settings did not affect TMATV repeatability. TTB repeatability was more robust and was not affected by changes in reconstruction settings, regardless of tracer uptake time or delineation method.

### Repeatability: impact of lesion delineation method

The delineation method had overall significant impact on the repeatability of both TMATV (*p* < 0.001) and TTB (*p* = 0.007). At 60 min post-injection, repeatability was significantly different whether delineations were performed with 41MAX or MV3 methods (TMATV TRT% 5.21% ± 2.58%, *p* = 0.044; TTB TRT% 3.78% ± 1.84%, *p* = 0.040), while for scans with 90 min of tracer uptake, A50P and MV2 provided significantly different TMATV repeatability (4.85% ± 2.39%; *p* = 0.042), regardless of reconstruction settings. EARL compliant reconstructions did not provide significantly different repeatability by the use of different delineation methods; however, with PSF reconstruction, it had impact on the repeatability of 41MAX as compared with MV3 (TMATV TRT% 3.06% ± 1.39%, *p* = 0.028; TTB TRT% 1.89% ± 0.90%, *p* = 0.036), regardless of tracer uptake time. Additionally, at a complete specification of tracer uptake interval and reconstruction settings, only 41MAX compared with MV3 had significantly different TMATV repeatability (6.65% ± 3.38%; *p* = 0.049; scan 60 min post-injection, EARL compliant reconstruction).

## Discussion

In the present work, we studied the repeatability of two whole-body metrics (TMATV and TTB) and how they vary as a function of tracer uptake interval, PET/CT image reconstruction settings, and tumour delineation method. We found that the delineation performed by the consensus method MV2 was more reliable than any other standard PET-based semi-automatic segmentation method included in this study (JI = 0.7). However, the best repeatability was obtained with 41MAX (RC = 11% for TTB from EARL compliant image and scan 90 min after injection). MV2 had its best repeatability for TMATV under the aforementioned settings with RC = 15%.

One important aspect to address regarding semi-automatic delineation methods is their concordance with a segmentation that would be performed by an expert observer. In this study, the reference delineation was a consensus between three expert observers. Figure 2 shows that the data from RD falls in between the values acquired by the four standard PET-based semi-automatic methods. From that, it can be expected that a consensus method would coincide with RD, which is what is seen in Table 2, where MV2 has the highest JI for both days (JI = 0.7). Furthermore, MV2 had the smallest interquartile range of all methods, showing its reliability to provide a good segmentation regardless of the patient’s condition. It might be considered that a JI = 0.7 is not sufficiently high to be defined as a reliable method; however, it is important to notice that the approach for creating the reference delineation used on the current study is far from the daily clinical routine (i.e. three observers assessing each image), in addition to the high inter-observer variability they presented. Furthermore, previous studies [34, 35] suggested that different lesion delineation methods had similar prognostic value for progression-free survival and overall survival accuracy, at least in the context of lymphoma patients, despite the large difference in MATV resulting from these different methods. These studies highlight that despite possible technical and conceptual flaws of basic PET-based lesion delineation methods, they are still successful prognostic factors. Therefore, not necessarily the actual accuracy of segmentation but good reliability and reproducibility might be of more importance in a diagnostic or prognostic setting (not in a radiotherapy setting).

Table 3 shows that the repeatability of the expert delineations is improved by taking their consensus. However, even this RD’s TMATV repeatability showed lower performance than the ones obtained in any of the semi-automatic methods (Table 4). Although RD’s TTB repeatability was better than the semi-automatic delineation methods (for 60 min of tracer uptake scans reconstructed with EARL compliant settings), Table 3 shows that the individual observer repeatability is highly variable, highlighting the strong dependence on the observer for a reliable assessment, while semi-automatic delineation methods are observer independent. Nevertheless, the TMATV repeatability obtained with the semi-automatic delineation methods was not significantly different than those obtained by the semi-automatic delineation methods (Additional file 1: Table S4).

The segmentations’ reliability obtained in this study is in line with previous work performed by Schaefer et al. [28], where the performance of the consensus method was investigated at a lesion level. That study found that consensus approaches never provided the worst delineation when compared to its reference. In the present study, MV2 and MV3 never had the lowest JI (Fig. 3) and MV2 had the lowest JI interquartile range for both scan days (Table 2).

Kramer et al. [15] had previously reported a repeatability coefficient (from TRT%) of 31% for metabolic active tumour volume (MATV) and 24% for TLG (scan 90 min post-injection, EARL compliant reconstruction, and A50P delineation method), metrics analogous to the ones in the current study. Such results are either worse (MATV) or on par (TLG) with most RC% obtained in the current study from the semi-automatic delineation methods. Kramer et al. additionally assessed repeatability when using the PERCIST averaged criteria to select lesions (the PERCIST criteria selects only up to the five hottest lesions [36], and their uptake was averaged into a single value) and achieved repeatability coefficients of 13% for MATV and 10% for TLG, reaching values comparable to the best ones found in the current study, where we specifically studied whole body metrics. Comparing our results with those seen by Kramer et al., we therefore suggest that good repeatabilities can be obtained for NSCLC whole body metrics, as long as either 41MAX or MV2 are used for lesion delineation.

Other lung cancer studies reported repeatability based on the absolute difference between the repeated measurements [29, 37]. Nakamoto et al. [37] reported the standard deviation of the measured repeatability, and by multiplying it by 1.96, it is possible to estimate RC from that study. Therefore, a tumour volume repeatability with (estimated) RC = 5.0% was found. Furthermore, Nakamoto et al. also studied a metric similar to TTB, namely effective glycolic volume (product between the voxel volume and its SUV, then summed for all of the lesion’s voxels), and found (estimated) RC = 16% (scan 50–60 min post-injection, and tumour delineation based on a background adaptive method). From 60 min post-injection scans, the current study has lowest TMATV RC = 8.9% (PSF reconstruction; A50P delineation method) and TTB RC = 15% (PSF reconstruction; MV3 delineation method) from aTRT%. Nakamoto et al. found lower (estimated) RC for both tumour volume and burden, which might be a consequence of only selecting lesions larger than 2.0 cm in all three dimensions (as determined by CT), avoiding partial volume effects. Their method, therefore, does not include the total tumour load in the body, unlike ours.

Frings et al. [29] reported TMATV RC = 44% and for lesions larger than 4.2 mL, RC = 21.9% (scans 45–60 min post [^18^F]FDG injection; delineation at 41% of SUV_max_ adapted for background), inferred from aTRT%. In the current study, both lower and higher RC values from aTRT% were found, depending on the delineation method. The best TMATV repeatability found (from aTRT%) was RC = 8.9% (60 min post-injection scan, PSF reconstruction, A50P delineation method).

Consistent with the results seen by Kramer et al. [15], we also observed that, in general terms, TMATV and TTB repeatabilities were not affected by tracer uptake time and reconstruction settings, but for a few specific cases with certain delineation methods. As seen previously [14, 20, 38], both TMATV and TTB repeatabilities were, overall, significantly dependent on the applied delineation method.

The main limitation of this study is the small sample size, consisting of ten patients scanned in a single PET/CT system. Furthermore, only a single lesion type (NSCLC, including extra-thoracic lesions) was considered and it was not feasible to perform fully manual segmentation of lesions as reference. However, the strength of the data is that we could compare segmentation performance of several semi-automatic methods against a reference derived from three expert observers and in a head to head comparison across variously applied tracer uptake intervals and reconstruction settings.

In conclusion, this study suggests that for [^18^F]FDG PET/CT studies in advanced stage NSCLC patients, a consensus approach (MV2) provides the best trade-off between most reliable delineation and overall repeatability performance. Furthermore, the PET-based semi-automatic delineation methods used as input for MV2 are simple and readily available. Therefore, its implementation seems feasible in most centres. However, if this consensus approach cannot be made widely available or shared in multicentre setting, the 41MAX method is the best alternative, since it also provides reliable segmentations and has the lowest RC% across all methods tested. Yet, one should be aware that the actual TMATV and TTB values obtained depend on the segmentation (Fig. 2) and the used delineation method should thus be consistently applied by all sites.

## Conclusion

In this study, we assessed the repeatability of total metabolic active tumour volume and total tumour burden in stage 3 and 4 NSCLC patients as a function of tracer uptake interval, image reconstruction settings, and lesion delineation method. We showed that, in most cases, changes in these parameters do not significantly affect TMATV and TTB repeatability. The consensus approach, MV2, was the most robust for accurately segmenting lesions. Based on delineation reliability and overall TMATV and TTB repeatability performance, a consensus segmentation approach, based on the majority vote method, is the most preferred semi-automated method for total tumour burden assessments in NSCLC [^18^F]FDG PET/CT studies.

## Additional file

Additional file 1: **Table S1.** Jaccard index and interquartile range of the semi-automatic delineation methods. **Table S2.** Total Metabolic Active Tumour Volume absolute repeatability for different tracer uptake intervals, reconstruction settings, and lesion delineation methods. **Table S3.** Total Tumour Burden absolute repeatability for different tracer uptake intervals, reconstruction settings, and lesion delineation methods. **Table S4.** Comparison of the TMATV repeatability obtained by RD and the semi-automatic delineation methods. **Figure S1.** Example of delineations performed by the consensus between three experienced observers (Reference delineation), and six semi-automatic methods with contour at: fixed SUV threshold of 2.5 g/mL (SUV25), fixed SUV threshold of 4.0 g/mL (SUV40), at 41% of lesion’s maximum SUV (41MAX), contrast corrected for local tumour to background activity at 50% of peak SUV (A50P), agreement between two or more of the previous methods (MV2), and agreement between three or four of the previous methods (MV3). Image acquired 60 min post-injection and reconstructed following EARL compliant settings. (DOCX 4220 kb)

## Abbreviations

[^18^F]FDG: [^18^F]2-Fluoro-2-deoxy-2-D-glucose
41MAX: 41% of lesion’s maximum SUV
A50P: Contrast adapted at 50% of lesion’s peak SUV
aTRT: Absolute test-retest
aTRT%: Relative absolute test-retest
CT: Computed tomography
EARL: European Association of Nuclear Medicine Research Ltd.
GEE: Generalized estimating equations
ICC: Intraclass correlation coefficient
JI: Jaccard index
MATV: Metabolic active tumour volume
MV2: Agreement between 2, 3 or 4 of standard PET-based delineation methods
MV3: Agreement between 3 or 4 of standard PET-based delineation methods
NSCLC: Non-small cell lung cancer
PET: Positron emission tomography
PSF: Point-spread-function
RC: Repeatability coefficient
RD: Reference delineation
RD1: Reference delineation for day 1
RD2: Reference delineation for day 2
SD: Standard deviation
SUV: Standardized uptake value
SUV25: Fixed SUV threshold of 2.5 g/mL
SUV40: Fixed SUV threshold of 4.0 g/mL
TLG: Total lesion glycolysis
TMATV: Total metabolic active tumour volume
TRT: Test-retest
TRT%: Relative test-retest
TTB: Total tumour burden
