# Supplementary material for: Repeatability of [18F]FDG PET/CT total metabolic active tumour volume and total tumour burden in NSCLC patients
Source: EJNMMI Res. 2019 Feb 7;9:14. doi: 10.1186/s13550-019-0481-1 (PMC6367490; doi:10.1186/s13550-019-0481-1)
Supplement: Supplementary file 1 — Table S1. Jaccard index and interquartile range of the semi-automatic delineation methods. Table S2. Total Metabolic Active Tumour Volume absolute repeatability for different tracer uptake intervals, reconstruction settings, and lesion delineation methods. Table S3. Total Tumour Burden absolute repeatability for different tracer uptake intervals, reconstruction settings, and lesion delineation methods. Table S4. Comparison of the TMATV repeatability obtained by RD and the semi-automatic delineation methods. Figure S1. Example of delineations performed by the consensus between three experienced observers (Reference delineation), and six semi-automatic methods with contour at: fixed SUV threshold of 2.5 g/mL (SUV25), fixed SUV threshold of 4.0 g/mL (SUV40), at 41% of lesion’s maximum SUV (41MAX), contrast corrected for local tumour to background activity at 50% of peak SUV (A50P), agreement between two or more of the previous methods (MV2), and agreement between three or four of the previous methods (MV3). Image acquired 60 min post-injection and reconstructed following EARL compliant settings. (DOCX 4220 kb) [file 13550_2019_481_MOESM1_ESM.docx]

**Additional File 1**

Table S1: Jaccard Index and interquartile range of the semi-automatic delineation methods

| Delineation Method | 60 minutes of tracer uptake time | | | | | | | |
| --- | --- | --- | --- | --- | --- | --- | --- | --- |
|  | EARL reconstruction | | | | PSF reconstruction | | | |
|  | Day 1 | | Day 2 | | Day 1 | | Day 2 | |
|  | Average JI | Interquartile Range | Average JI | Interquartile Range | Average JI | Interquartile Range | Average JI | Interquartile Range |
| SUV25 | 0.62 | [0.48 – 0.73] | 0.55 | [0.39 – 0.72] | 0.62 | [0.44 – 0.74] | 0.49 | [0.37 – 0.62] |
| SUV40 | 0.62 | [0.54 – 0.77] | 0.59 | [0.46 – 0.80] | 0.62 | [0.59 – 0.69] | 0.62 | [0.50 – 0.86] |
| 41MAX | 0.65 | [0.45 – 0.88] | 0.64 | [0.45 – 0.85] | 0.55 | [0.40 – 0.81] | 0.55 | [0.40 – 0.75] |
| A50P | 0.50 | [0.37 – 0.65] | 0.50 | [0.35 – 0.67] | 0.49 | [0.38 – 0.67] | 0.50 | [0.35 – 0.63] |
| MV2 | 0.71 | [0.61 – 0.80] | 0.70 | [0.54 – 0.86] | 0.69 | [0.61 – 0.77] | 0.69 | [0.54 – 0.86] |
| MV3 | 0.58 | [0.39 – 0.77] | 0.56 | [0.40 – 0.79] | 0.51 | [0.40 – 0.66] | 0.53 | [0.39 – 0.65] |
|  | 90 minutes of tracer uptake time | | | | | | | |
|  | EARL reconstruction | | | | PSF reconstruction | | | |
|  | Day 1 | | Day 2 | | Day 1 | | Day 2 | |
|  | Average JI | Interquartile Range | Average JI | Interquartile Range | Average JI | Interquartile Range | Average JI | Interquartile Range |
| SUV25 | 0.44 | [0.35 – 0.54] | 0.46 | [0.34 – 0.59] | 0.44 | [0.35 – 0.54] | 0.44 | [0.33 – 0.60] |
| SUV40 | 0.48 | [0.44 – 0.59] | 0.47 | [0.40 – 0.56] | 0.49 | [0.43 – 0.61] | 0.48 | [0.39 – 0.56] |
| 41MAX | 0.48 | [0.34 – 0.65] | 0.47 | [0.37 – 0.49] | 0.42 | [0.28 – 0.53] | 0.42 | [0.29 – 0.49] |
| A50P | 0.40 | [0.28 – 0.44] | 0.40 | [0.30 – 0.48] | 0.35 | [0.23 – 0.40] | 0.34 | [0.27 – 0.42] |
| MV2 | 0.53 | [0.46 – 0.64] | 0.55 | [0.42 – 0.63] | 0.53 | [0.48 – 0.61] | 0.55 | [0.41 – 0.63] |
| MV3 | 0.44 | [0.32 – 0.54] | 0.44 | [0.37 – 0.49] | 0.39 | [0.24 – 0.43] | 0.36 | [0.29 – 0.43] |

Average Jaccard Index (JI) and the interquartile range of each semi-automatic delineation method when compared with the reference delineation of each day. Semi-automatic methods applied to images acquired 60 minutes post-injection on the top section of the table and 90 minutes post-injection in the bottom section. Images reconstructed with EARL compliant settings on the left part of the table and reconstructed with PSF settings on the right section of the table.

Table S2: Total Metabolic Active Tumour Volume absolute repeatability for different tracer uptake intervals, reconstruction settings, and lesion delineation methods.

|  | 60min of uptake | | 90min of uptake | |
| --- | --- | --- | --- | --- |
| Method | aTRT (RC) | aTRT% (RC%) | aTRT (RC) | aTRT% (RC%) |
| EARL |  |  |  |  |
| SUV25 | 44.1 (200) | 14.6 (43) | 15.4 (23) | 6.2 (11) |
| SUV40 | 12.3 (36) | 11.1 (22) | 7.7 (9.6) | 10.3 (29) |
| 41MAX | 12.4 (25) | 14.9 (37) | 9.0 (27) | 5.8 (10) |
| A50P | 12.2 (28) | 16.4 (38) | 16.5 (77) | 10.9 (36) |
| MV2 | 17.5 (38) | 15.5 (36) | 9.2 (18) | 5.7 (12) |
| MV3 | 12.5 (27) | 14.5 (26) | 18.3 (76) | 11.9 (35) |
| RD | 28.6 (59) | 23.0 (43) |  |  |
| PSF |  |  |  |  |
| SUV25 | 49.6 (167) | 17.0 (40) | 145 (733) | 16.7 (60) |
| SUV40 | 9.0 (16) | 10.5 (20) | 7.9 (13) | 7.7 (19) |
| 41MAX | 5.4 (14) | 10.9 (30) | 20.8 (89) | 11.2 (23) |
| A50P | 5.4 (11) | 8.0 (8.9) | 7.9 (15) | 10.5 (15) |
| MV2 | 9.9 (18) | 9.7 (29) | 18.4 (71) | 7.5 (19) |
| MV3 | 5.2 (11) | 7.7 (11) | 7.4 (15) | 9.3 (12) |

Average (and repeatability coefficient = 1.96×SD) of Total Metabolic Active Tumour Volume (TMATV) absolute repeatability for different tracer uptake intervals, reconstruction settings, and lesion delineation methods. Difference is in mL. Relative difference is a percentage.

Table S3: Total Tumour Burden absolute repeatability for different tracer uptake intervals, reconstruction settings, and lesion delineation methods.

|  | 60min of uptake | | 90min of uptake | |
| --- | --- | --- | --- | --- |
| Method | aTRT (RC) | aTRT% (RC%) | aTRT (RC) | aTRT% (RC%) |
| EARL |  |  |  |  |
| SUV25 | 405 (1475) | 18.4 (43) | 122 (229) | 7.8 (11) |
| SUV40 | 315 (1066) | 19.5 (36) | 96 (170) | 13.0 (27) |
| 41MAX | 162 (569) | 17.4 (36) | 72 (145) | 5.3 (5.5) |
| A50P | 191 (597) | 22.6 (35) | 114 (251) | 13.2 (31) |
| MV2 | 324 (1057) | 20.3 (39) | 101 (171) | 8.6 (13) |
| MV3 | 165 (568) | 17.7 (32) | 104 (262) | 10.7 (32) |
| RD | 164 (384) | 16.5 (28) |  |  |
| PSF |  |  |  |  |
| SUV25 | 375 (975) | 17.1 (34) | 521 (2438) | 13.3 (37) |
| SUV40 | 214 (700) | 15.9 (22) | 101 (173) | 10.5 (19) |
| 41MAX | 101 (285) | 12.7 (28) | 89 (263) | 8.0 (19) |
| A50P | 113 (294) | 13.5 (16) | 83 (145) | 11.9 (22) |
| MV2 | 215 (700) | 14.7 (28) | 131 (240) | 9.9 (17) |
| MV3 | 100 (278) | 11.6 (15) | 51 (101) | 6.7 (14) |

Average (and repeatability coefficient = 1.96×SD) of Total Tumour Burden (TTB) absolute repeatability for different tracer uptake intervals and lesion delineation methods. Difference is in grams. Relative difference is a percentage. ICC of the repeated measurements is also shown.

**Table S4:** Comparison of the TMATV repeatability obtained by RD and the semi-automatic delineation methods.

| Repeatability of | Mean difference (μ) | Standard error (σ) | Significance (p) |
| --- | --- | --- | --- |
| RD *versus* SUV25 | -15.6% | 14.2% | 0.270 |
| RD *versus* SUV40 | -10.7% | 12.7% | 0.397 |
| RD *versus* 41MAX | -17.6% | 14.1% | 0.211 |
| RD *versus* A50P | -15.6% | 13.6% | 0.249 |
| RD *versus* MV2 | -19.1% | 13.6% | 0.160 |
| RD *versus* MV3 | -10.9% | 13.7% | 0.423 |

Mean difference, standard error, and significance (from the GEE analysis) comparing the TMATV repeatability of RD with the six semi-automatic delineation methods (data from 60 minutes post-injections scans reconstructed with EARL compliant settings). The GEE model analysis was setup so that TMATV TRT% was included as dependent variable and the delineation methods were considered predictors (i.e. independent variables) for the model, the remaining settings follow the description on the main text.


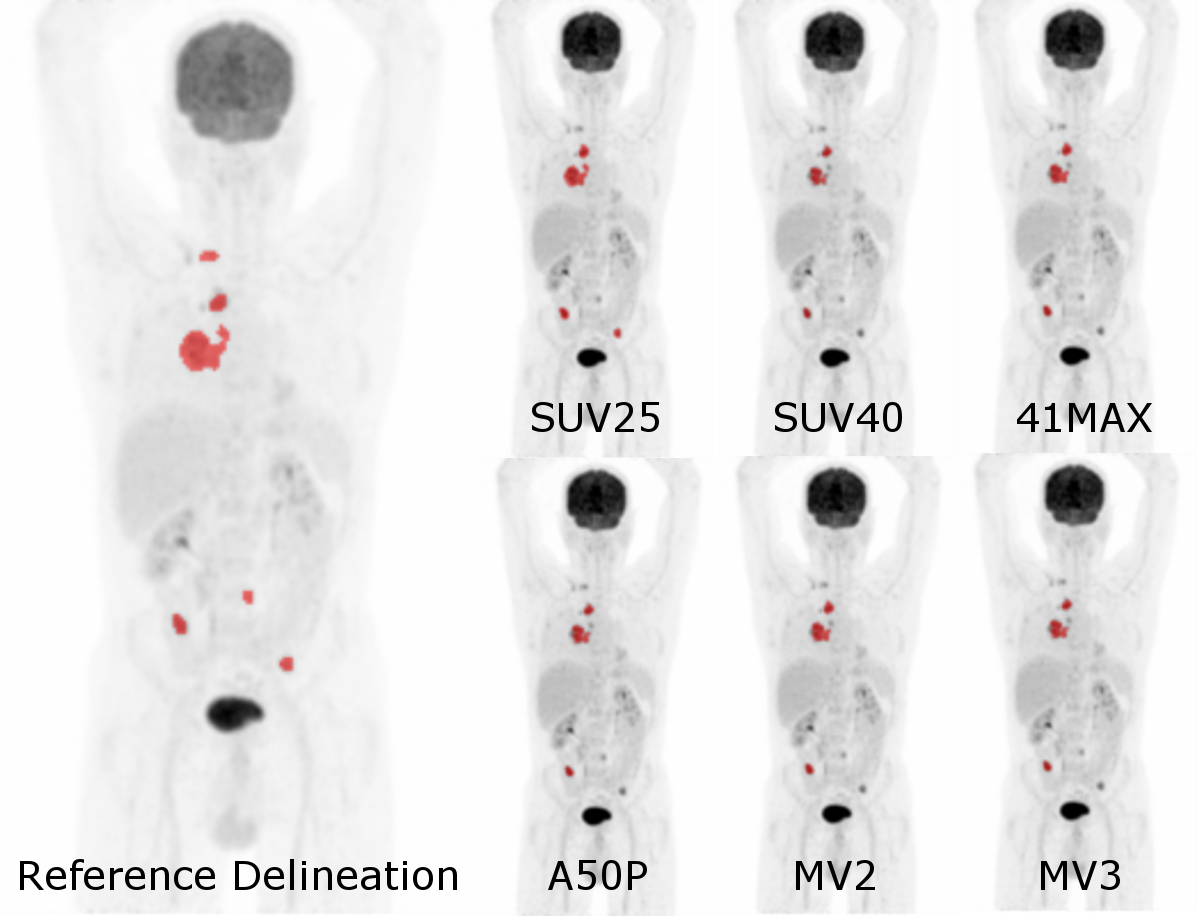


Figure S1: Example of delineations performed by the consensus between three experienced observers (Reference delineation), and six semi-automatic methods with contour at: fixed SUV threshold of 2.5 g/mL (SUV25), fixed SUV threshold of 4.0 g/mL (SUV40), at 41% of lesion’s maximum SUV (41MAX), contrast corrected for local tumour to background activity at 50% of peak SUV (A50P), agreement between two or more of the previous methods (MV2), and agreement between three or four of the previous methods (MV3). Image acquired 60 minutes post-injection and reconstructed following EARL compliant settings.
